# Porous SnO_2_ nanoparticles based ion chromatographic determination of non-fluorescent antibiotic (chloramphenicol) in complex samples

**DOI:** 10.1038/s41598-018-29922-5

**Published:** 2018-08-17

**Authors:** Nadeem Muhammad, Abdul Rahman, Muhammad Adnan Younis, Qamar Subhani, Khurram Shehzad, Hairong Cui, Yan Zhu

**Affiliations:** 1 Department of Environmental Engineering, Wuchang University of Technology, Wuhan, China; 2 Department of Chemistry, Zhejiang University, Hangzhou, 310028 China; 3 Department of IT and Electronics, 310027 Hangzhou, China

## Abstract

Nowadays, there are rising concerns about the extensive use of the antibiotics such as chloramphenicol (CAP), has threatened the human life in the form of various vicious diseases. The limited selectivity and sensitivity of confirmatory techniques (UV and electrochemical) and non-fluorescence property of CAP make its determination a challenging task in the modern pharmaceutical analysis. In order to redeem the selective, sensitive and cost-effective fluorescence methodology, here by the dual role of synthesized porous SnO_2_ nanoparticles were exploited; (i) a porous sorbent in a µ-QuEChERS based sample preparation and as (ii) a stimulant for the transformation of non-fluorescent analytes namely CAP and p-nitrophenol (p-NP) into their respective fluorescent product. We report a green, simple, selective and cost effective ion chromatographic method for CAP sensitive determination in three complex matrices including milk, human urine and serum. The synthesized sorbent not only selectively adsorbed and degraded the matrix/interferences but also selectively reduced the non-fluorescent antibiotic CAP into a fluorescent species. This developed ion chromatographic method exhibited good selectivity, linearity (r^2^ ≥ 0.996) and limit of detection (LOD) was in the range 0.0201–0.0280 µg/kg. The inter- and intraday precisions were also satisfactory having a relative standard deviation (RSDs) less than 14.96% and excellent recoveries of CAP in the range of 78.3–100.2% were retrieved in various complex samples.

## Introduction

Fenicol drugs family are synthetic and broad-spectrum antibiotics, which have been extensively consumed in livestock husbandry on large scale for treatment of various diseases^1,2^. The chloramphenicol (CAP) which is the first ever nitro compound that was found in nature^3,4^. Due to its unique characteristics low cost, long stability and ability to exhibit optimum activity in pH span of 7.4–8, has increased its expenditure as a bacteriostatic antibiotic for the treatment of various diseases in livestock and humans^5–7^. In addition, it is also widely used to enhance the meat amount during shrimp breeding^8^. However, due to its properties to bind with protein and quickly absorbed and distributed into the organ and tissues within hours have also entailed threatening effects on the health of human beings such as hypoplastic and aplastic anaemia, leukemia, blood dyscrasias bone marrow depression, DNA damage, gene toxicity, vomiting, nausea, allergic reactions, unpleasant taste, diarrhea, grey syndrome (cardiovascular collapse, respiratory depression, and coma) etc.^4,6,8–11^. The pregnant women and infants are particularly sensitive to its effects. Moreover, the emergence of drug-resistant bacteria and its toxic effects on the hemopoietic system have provoked the CAP banishment in the food chain and medication of animal diseases in various countries including USA, China, Canada, Poland, Iran and member states of European Union (EU). Therefore, minimum required performance limit (MRPL) for its determination was set as 0.30 μg/kg in animal origin food and the EU interim tolerance level for CAP residues was set as 10.0 µg/kg^3,10,12–16^. In spite of that, it’s great pharmacokinetics properties and low price have coerced its illegal use in livestock and aquaculture, especially in the developing countries^16,17^.

A number of chromatographic methodologies have been investigated for CAP determination after extracting it from different complex matrices by applying expensive, time consuming, tedious and non-environmental friendly extraction methods such as solid phase extraction (SPE)^18^, ultrasound-assisted dispersive liquid-liquid microextraction (DLLME)^19^, aqueous two-phase extraction system (ATPES)^20^ and QuEChERS sample preparation method^21,22^. However, the previous analytical techniques have various limitations; for examples, gas chromatography coupled with electron capture detector (ECD) is sensitive technique but requires complex chemical derivatization^23^, liquid chromatography coupled to mass spectrometry (LC-MS) is rapid and sensitive techniques but these instruments are too expensive for routine analyses and out of access in developing countries laboratories^5,24–26^. Unlike with UV, the LC coupled with fluorescence detector is a simple, cheap, highly selective and sensitive technique for analytes determination in complex samples but the non-fluorescence property of CAP has made its use limited^27,28^. Therefore, various indirect fluorescence methods i.e. derivatization^29^, florescent probe^30^, fluorescent nanosensor^31^ and photochemical induce detection were applied for its selective and sensitive in various complex matrices^32^. However, all above-mentioned techniques are complex, laborious and involved separate rigorous and expensive sample preparation methods. In order to reinstate cheap, selective and sensitive fluorescence techniques for CAP determination in complex matrices, green porous SnO_2_ nanoparticles were synthesized and their multiple roles were availed for the automatic transformation of both non-fluorescent CAP and p-NP into their respective fluorescence products, and at same time as a sorbent in a µ-QuEChERS based sample preparation method used for CAP extraction and maximum elimination of organic and inorganic matrix antagonism in three complicated samples.

Recently, a number of synthetic and commercial nano-materials (functionalized silica, multi-walled carbon nanotubes, graphene, magnetic and non-magnetic NPs) have been widely used as sorbents in various sample preparation techniques for enrichment, speciation and separation of different kind of analytes from various matrices^33–41^. In addition, there are increasing use of NPs in the synthesis of high-rate anode materials^42–46^, catalysis^47^, sensor^48^, solar cells^49^ and for the synthesis of HPLC stationary phase^50^.

The sorbent “SnO_2_ NPs” is unambiguously a better choice for this purpose as: (a) it need simple method of preparation; (b) require cost-effective starting materials; (c) it is nontoxic and not carcinogenic in nature; (d) it involve green synthesis and (e) its non-destructive nature to analyte of interest and high selectivity to reduce particular functional groups in the presence of other at that particular condition make it unique over other metal NPs^51^.

Therefore, porous SnO_2_ NPs were synthesized by following modified method reported by A. Bhattacharjee *et al*.^52^ and used in IC for sensitive and selective determination of CAP in three complex samples. It was observed SnO_2_ NPs size, porosity and morphology have a significant effect on their application as a porous sorbent in the µ-QuEChERS based sample preparation for matrices interference (organic and inorganic) elimination and for the transformation of the non-fluorescent CAP into its fluorescent product. The nanoparticles definite morphology and size were harnessed by the addition of an optimized amount of stabilizing (sodium dodecyl sulfate) and a capping agent (glycine)^41,52,53^.

Here, very first time dual application of porous SnO_2_ NPs as a sorbent for the matrices (polar interferences, dyes, heavy metals) selective degradation, adsorption and selective transformation of non-fluorescent antibiotic CAP into fluorescent species were exploited, which later cleanly separated and sensitively determined by using IC-FLD in three complex matrices including milk, human serum and urine.

## Results and Discussion

### Characterization of SnO_2_ nanoparticles

**Figure 1:**
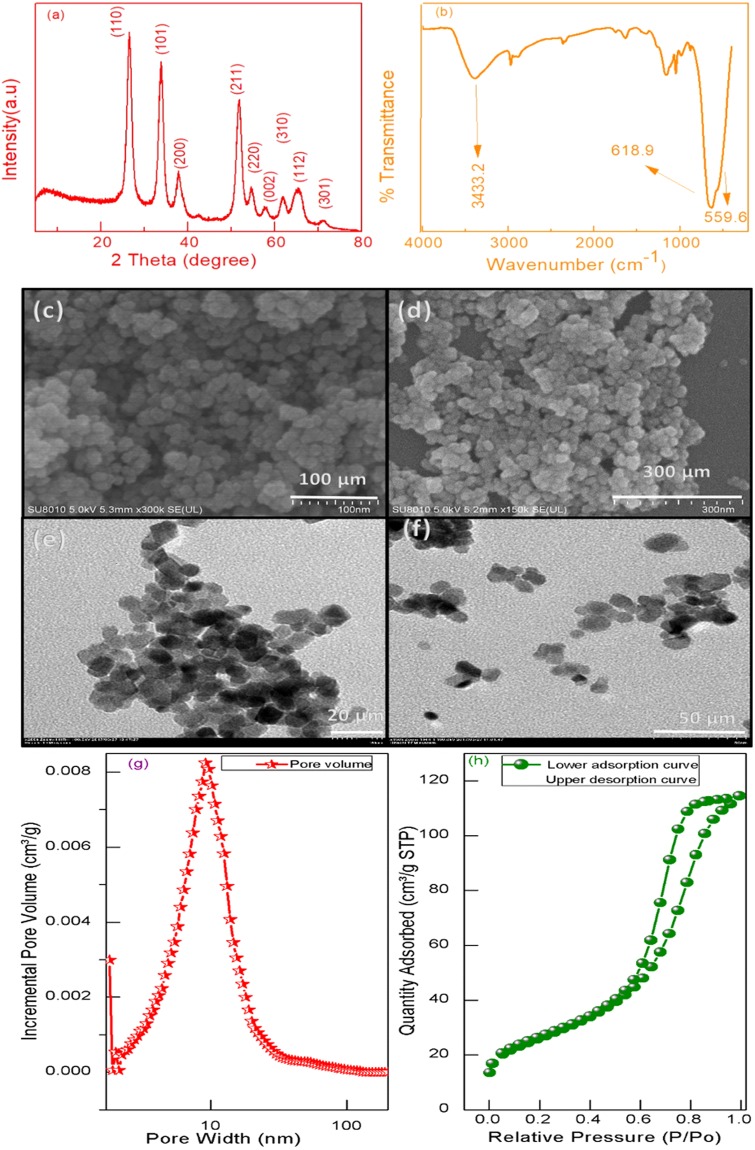
(**a**) XRD pattern of synthesized SnO_2_ nanoparticles; (**b**) FT-IR spectra of SnO_2_ nanoparticles formed at 600 °C and (**c**,**d**) SEM, (**e**,**f**) TEM images at different resolution of nanoparticles, (**g**) The corresponding BET surface areas and pore volume distributions of the SnO_2_ NPs and (**h**) N2 adsorption/desorption isotherms of the SnO_2_ NPs.

The structure and phase composition of synthesized SnO_2_ nanoparticles was characterized by XRD measurements. Figure 1a shows the peaks at 2 $\theta$ = 26.8°, 34.13°, 39.28°, 52.05°, 55.04°, 58.13°, 62.23°, 65.01°, and 66.18° which corroborate to the respective lattice plane (110), (101), (200), (211), (220), (002), (310), (112) and (301). These found position of peaks completely matched with the tetragonal rutile structure of SnO_2_ nanoparticles reported in the literature. No definite peaks were observed for other impurities, confirming the pure tetragonal rutile structure of SnO_2_ nanoparticles^52^.

Infrared characterization of the porous SnO_2_ nanoparticles was also performed in the range of 400 to 4000 cm^−1^ as depicted in Fig. 1(b). The peaks between 660–610 cm^−1^ are assigned to the Sn-O-Sn stretching vibration mode of surface bridging of SnO_2_ NPs, while the peak at 530–560 cm^−1^ is attributed due to the terminal Sn-O stretching vibration mode of Sn-OH group. The existence of –OH group is also confirmed by the peak appeared around 3433 cm^−1^. A strong peak around 618 cm^−1^ is an indication of potential conversion of stannous hydroxide into stannous oxide efficient at 600 °C, which is in agreement with the previous studies^52,54,55^.

$${\rm{Sn}}\mbox{--}{\rm{OH}}+{\rm{Sn}}\mbox{--}{\rm{OH}}\to {\rm{Sn}}\mbox{--}{\rm{O}}\mbox{--}{\rm{Sn}}+{\rm{H}}-{\rm{O}}-{\rm{H}}$$

Size distribution and morphology study of SnO_2_ NPs was carried out by SEM, TEM images and BET method as shown in Fig. 1a(c-h). Both SEM and TEM images show the good dispersion of synthesized SnO_2_ nanoparticles with mean diameter of about 11.92 nm (RSD = 2.09%) which was calculated from fifty random NPs (see Fig. S1). The TEM images also show the spherical and polycrystalline nature of SnO_2_ NPs which are composed of individual nanocrystallites. The spherical shape SnO_2_ nanoparticles under the overlapped nanospheres can be visibly observed, which indicates the rough surface and porosity of NPs, which enhances their matrices adsorption and electron conducting capabilities^56,57^. The SnO_2_ NPs showed an average pore size in mesopore range between 6.02 to 7.6 nm at 600 °C, whereas their pore volume about 0.177068 cm³/g was observed as shown in Fig. 1(g). The large pore size help to adsorb and reduce maximum CAP into its fluorescent specie and meanwhile these pore volume also help to adsorb matrix interferences to avoid matrix effect. This phenomenon can be observed from N_2_ adsorption/desorption isotherms of SnO_2_ NPs, indicating the presence of mesopores of adsorption and desorption as shown in Fig. 1(h).

### Scanning of the fluorescent species (CAP) and its IC separation

The reduced CAP solution was collected and subsequently scanned with a fluorescence detector in alkaline media and λ_em_/λ_ex_ = 232/361 nm was observed as its optimum wavelength for sensitive fluorescence detection as displayed in Fig. S2.

**Figure 2:**
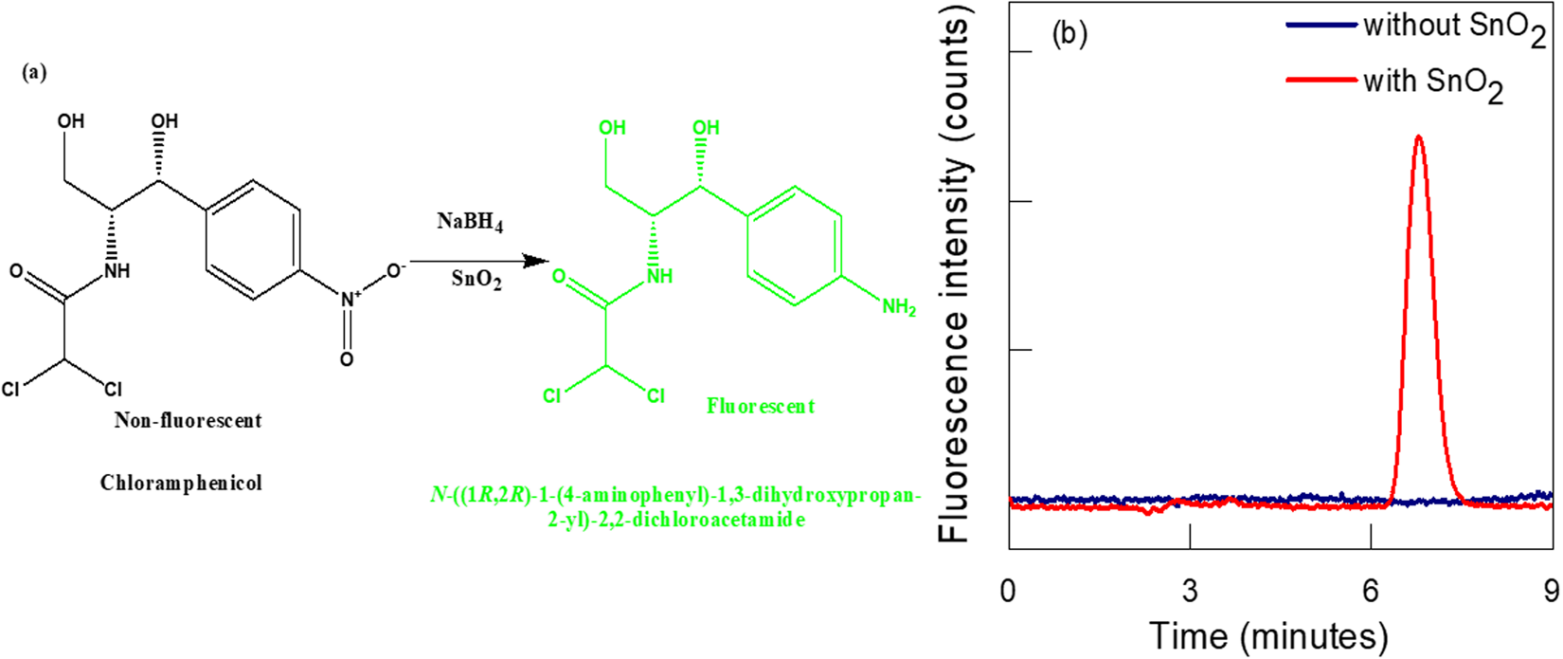
(**a**) The chemical conversion of CAP into a fluorescent product; and (**b**) The fluorescence intensity of CAP in basic media with (red line) and without (blue line) sorbent treatment.

A clean isocratic separation of CAP was performed by using a regular anion-exchange IonPac^®^ AS12 column protected with IonPac^®^ AG 12 guard column. The mobile phase 35 mM NaOH with only 15% acetonitrile at a normal flow rate of 1.0 mL/min was selected, which elute CAP at 7.1 min as given in Table 1. The isocratic elution of standard solution of CAP (1 mg/kg) with and without treatment of SnO_2_ sorbent is presented in Fig. 2(a,b). It shows that that CAP can only be detected after SnO_2_ sorbent treatment. The fluorescent property of reduced CAP can be attributed due to the conversion of electron withdrawing –NO_2_ into electron donating –NH_2_ group, which results in electron delocalization to an aromatic ring. Secondly, the alpha proton also ionized in basic media, which further helps to stabilize the resonating electrons and gave high fluorescence intensity.

**Table 1:** The analytical figure of merit of CAP in pure solvent.

Analyte	Linearity range (mg/kg)	Correlation coefficient (r^2^)	LOD (ug/kg)	LOD (ug/kg)	Precision (RSD, %)		Retention time (min)
					Intra	Inter	
CAP	0.01–5	0.997	0.028	0.093	5.62	8.69	7.1

The reduction mechanism of CAP into a fluorescent species involved the following two steps: (a) transfer of electrons to the CAP nitro functional group and (b) protonation.

**Figure 3:**
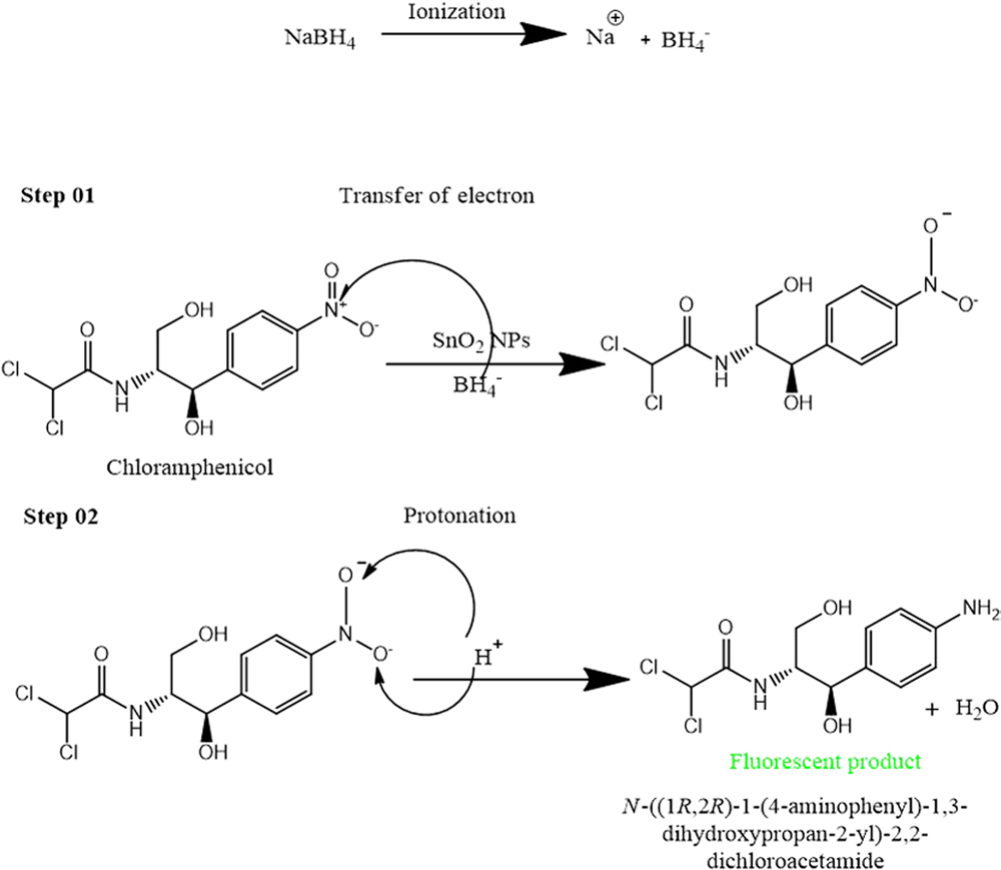
Mechanism of the reduction of CAP into a fluorescent specie.

Both CAP and *p*-NP reduction mechanism involved the transfer of electrons from the donor BH_4_^−1^ ions, while SnO_2_ NPs presence in the solution help to adsorb BH_4_^−1^ ions and discharge of electrons from BH_4_^−1^ to the receptor species i.e. CAP or *p*-NP. In the second step, the aqueous protic solvent facilitates the required amount of H^+^ ion for the maximum reduction of CAP/*p*-NP into their respective fluorescent species as the detailed mechanism is given in Fig. 3.

### Optimization of factors affecting the fluorescence intensity of CAP

There are various parameters which directly or indirectly influenced the fluorescence intensity of CAP. Effect of these parameters were investigated and optimized such as optimization of sorbent amount, optimization of NaBH_4_ concentration, optimization of sonication time, effect of agitation temperature and evaluation of extracting solvent.

The flexibility of µ-sample preparation is very important for the analysis of particular analyte of interest in various matrices. Therefore, herein synthesized porous SnO_2_ nanoparticles were used as sorbent and their dual application were exploited in µ-sample preparation for the transformation of the non-fluorescent CAP into the fluorescent product and to retain and degrade matrices/interferences at the same time. It also helped to transfer the analyte of interest in the organic phase.

**Figure 4:**
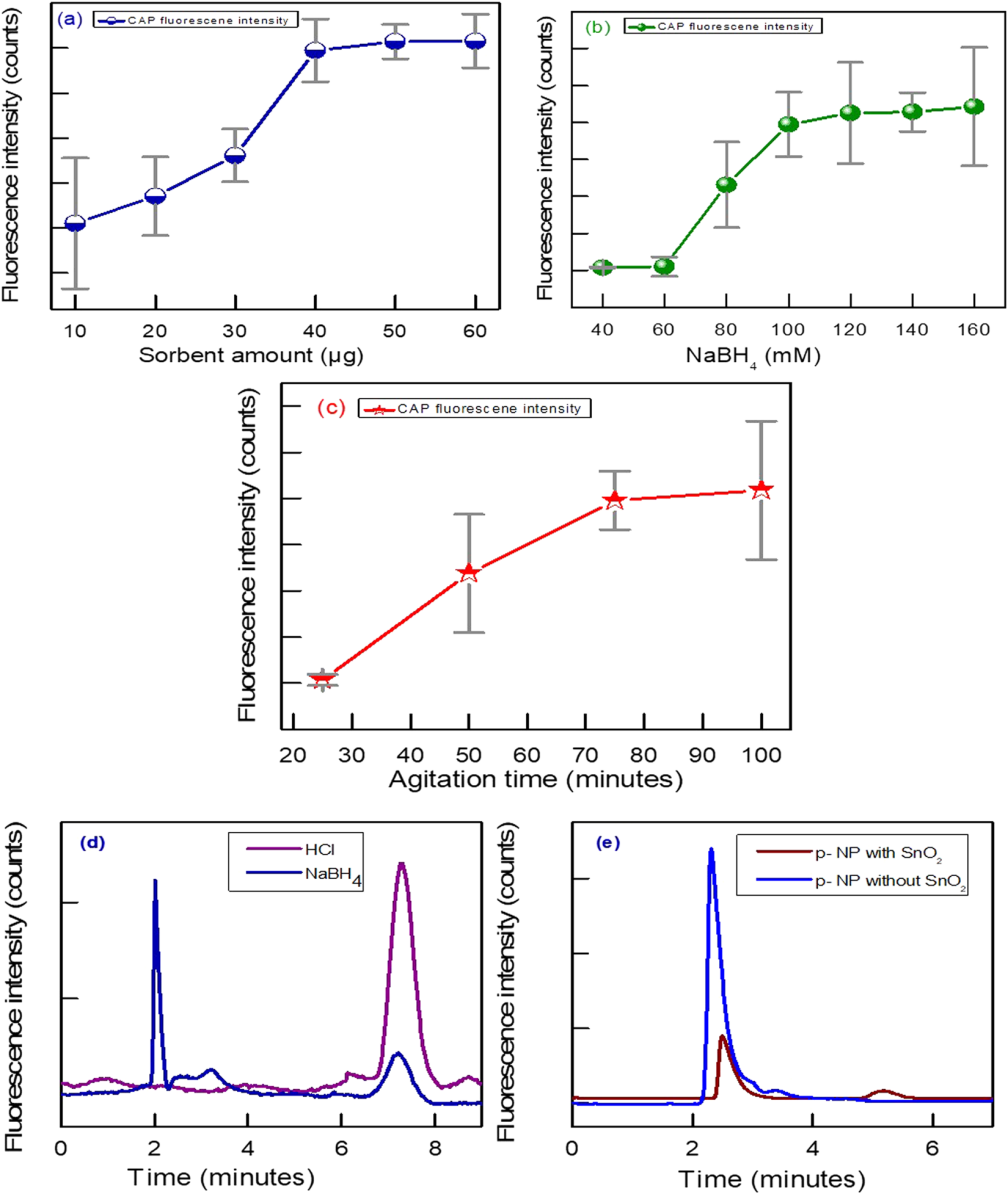
(**a**) Effect of different amount of sorbent on CAP fluorescence intensity; (**b**) effect of NaBH4 concentration on CAP fluorescent intensity; (**c**) effect of agitation time on fluorescent intensity of CAP; (**d**) comparison of effect of two reducing agent (NaBH4 and HCl) on fluorescence intensity of CAP and (**e**) Conversion of non-fluorescent p-nitro phenol into fluorescent specie after sorbent treatment.

During µ-sample preparation and optimization, it was observed that different amount of sorbent has a significant influence on interferences elimination and analyte recovery in different extracts. Therefore, different amount of sorbent was varied from (10, 20, 30, 40, 50 and 60 µg) and analytes recoveries were investigated. Herein, the fluorescent peak of analyte starts appearing at 30 µg sorbent and reached to maximum at 40 µg. Afterward, no significant increase was observed in fluorescence intensity of CAP in standard solution as shown in Fig. 4(a,b). Interestingly, during different samples analysis, it was observed the maximum recovery of analytes and low matrix peak appeared at 50 µg of the sorbent amount as it can be observed in sample chromatogram Fig. 4(a).

Hence, in order to utilize, dual application of sorbent 50 µg was chosen and used for further experiments to have maximum fluorescence quantum yield and recoveries of CAP in three samples.

Sodium borohydride is a reducing agent and its various advantages were exploited in this study. For example, (a) decomposition of various organic matrices pigments and dyes particularly, (b) reductive transformation of the non-fluorescent CAP into a fluorescent product and (c) enhancement of the extraction recovery by reducing opposite force of attraction between CAP antibiotic and matrices component. Its effect on the activity of sorbent was investigated by comparing CAP fluorescent signal intensity at various NaBH_4_ (40, 60, 80, 100, 120, 140 and 160 mM) concentration. It was observed the fluorescent signal appeared when NaBH_4_ concentration reached at 0.08 M and became maximum at 0.1 M, after that there was not any reproducible and significant increase in the fluorescent intensity of CAP as it can be observed from Fig. 4(b). It shows that 0.1 M NaBH_4_ is enough to reduce maximum CAP into a fluorescent specie. HCl was also used in place of NaBH_4_ for the reduction of the non-fluorescent CAP into a fluorescent specie. But the peak area of the fluorescent CAP was reduced significantly in the presence of HCl, this might be due to the fluorescence quenching effect of Cl^−1^ ions. Effect of both the reducing agents on CAP peak area can be observed from the Fig. 4(d).

In this µ-extraction ultra-sonic agitation is immensely vital for extraction of analytes of interest from various matrices, especially when its multi-purpose uses were exploited: for conversion of the non-fluorescent CAP into a fluorescent specie (chemical reduction process), extraction of CAP from various complex matrices and for acceleration of analyte mass transfer into the organic phase. The ultra-sonication time was varied and it was observed the CAP fluorescent peak appear after 30 minutes and continues to increase with the passage of time and became maximum at 75 minute, which reflects the maximum conversion of CAP into a fluorescent species, as shown in Fig. 4(c). This elongated time also enough to overcome the need for extra time which is normally required for analytes extraction from various complex matrices. Therefore, CAP maximum recoveries were obtained in the range 88.6–100.2% in various matrices as given in Table 2. This time also appearsed large enough for adsorption and degradation of different matrices interferences and impurities under sunlight^52^.

**Table 2:** Linearity, limit of detection, precision, accuracy, recovery and matrix effect in various samples for CAP obtained by IC-FLD.

Sample	Linearity range (mg/kg)	Correlation coefficient (r^2^)	LOD (µg/kg)	Precision (RSD, %)		Matrix effect (%)
				Intra	Inter	
Milk	0.01–5	0.097	0.023	5.03	14.85	92.3 (4.78)
Serum	0.01–5	0.098	0.020	13.78	9.34	101.68 (9.87)
Urine	0.01–5	0.099	0.027	2.03	8.96	96.03 (5.45)

(n = 5).

Contrary to the other extraction methods, the temperature is regarded as an important parameter in this particular µ-QuEChERS based sample preparation for the chemical reduction of CAP into a fluorescent specie. It was observed that more efficient conversion of the non-fluorescent CAP into a fluorescent CAP occurs above 40 °C and gave a high fluorescent peak at 60 °C. However, with further increase of temperature abnormal peak start appearing at R_t_ 3.1 minute. It shows the possible chances of decomposition of CAP into more than one fluorescent products and this abnormal peak started to become high with the increase of temperature as shown in Fig. S3(a). Therefore, more suitable temperature 50–55 °C was chosen for further experiments.

It is also extremely vital to select an appropriate solvent in order to have a maximum recovery of desire analyte and to guarantee the minimal co-extraction of impurities and interferences. Various solvents including methanol, acetonitrile, acetone and dichloromethane were evaluated for CAP extraction from complex samples. Among these extraction solvents, acetonitrile exhibited desired results as its interaction toward organic analytes of interest to dissolve them quickly and its ability of back extraction of dissolved polar interfering impurities and matrices into aqueous phase via “salting-out” effect^54,58^. While methanol not only have dissolved the matrices and impurities but also was unable to form two layers. Therefore, huge positive matrix effect was observed. The other used solvents exhibited a poor recovery of the analyte. The effect of these four solvents on CAP recovery can be observed from Fig. S3(b).

### Analysis of milk, human urine and serum samples

**Figure 5:**
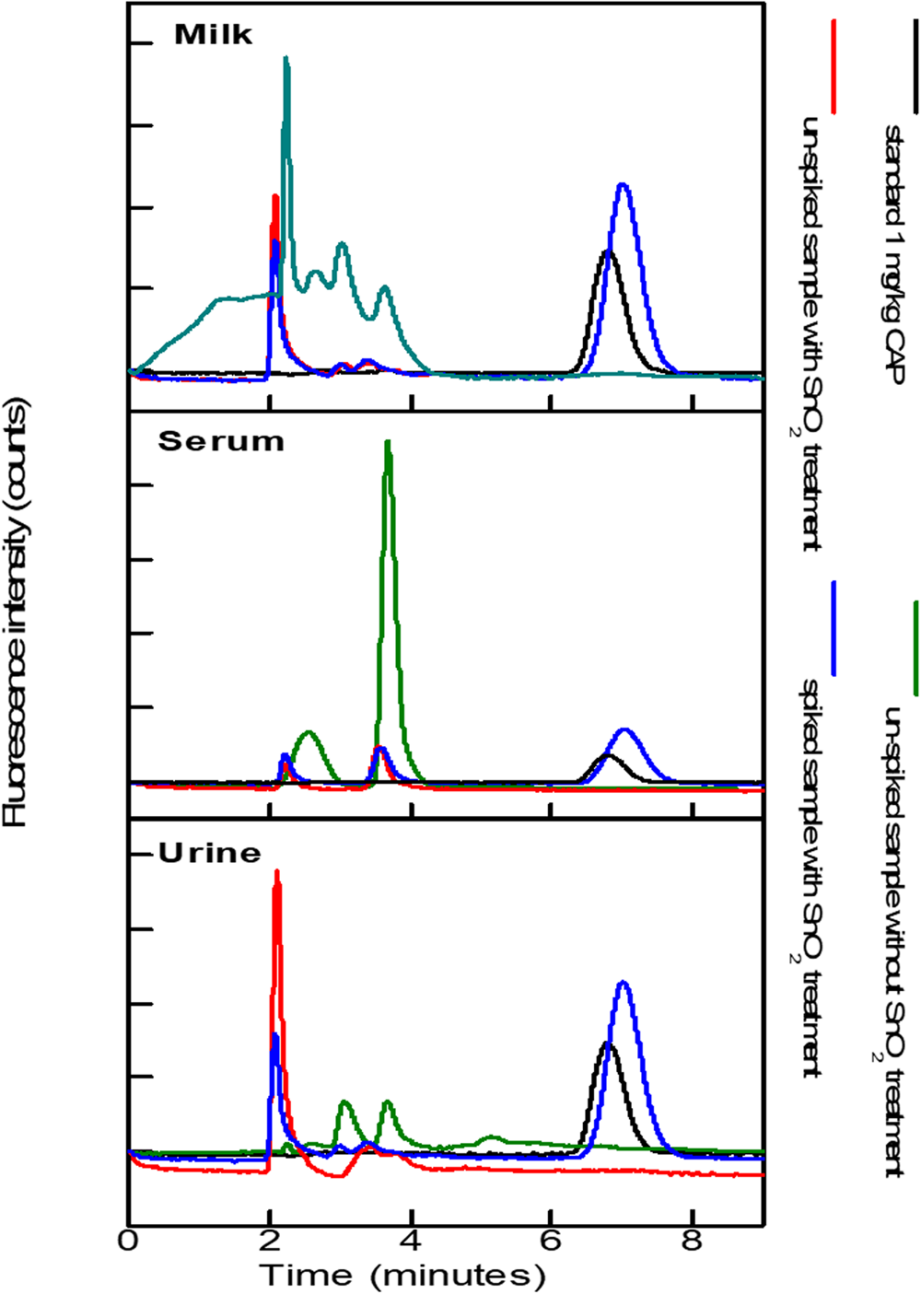
Samples (milk, human serum and urine) chromatograms spiked with CAP (0.48 mg/kg) after extraction with µ-sample extraction method: (black line) standard with sorbent treatment; (green line) real sample after without sorbent treatment; (red line) real sample after sorbent treatment and (blue line) spiked sample after sorbent treatment.

Applications of this developed method was investigated for the analysis of antibiotic CAP in three diverse complex matrices including human urine, milk, and serum. The clean ion chromatographic based isocratic separation and extremely sensitive fluorescence detection of CAP in complex samples is displayed in Fig. 5(a–c).

### Validation of Method

The IC-FLD chromatograms of blank and spiked milk, human serum and urine samples are shown in Fig. 5(a–c), which exhibit good selectivity as there is no interfering peak at the retention time of CAP. The excellent linearity was gained in the range 0.48–5 mg/kg. The LOD determined as the minimum CAP concentration showing a signal to noise ratio of 3 was 14.6 µg/kg, whereas the LOQ was 48 µg/kg as depicts in the following Tables 1 and 2.

The intra- and inter-day precisions are represented in term of relative standard deviations (RSDs). Both including intra-day recoveries and precisions were determined by anatomizing all three fortified samples in quintet per day at two fortification levels 0.48 and 5.0 mg/kg, while the inter-day recoveries and precisions were obtained by running the fortified samples for two successive days. At both fortified levels, RSDs of intra- and inter-day were in the range of 0.55–14.7% and 4.8–15.98%, respectively. The recoveries of CAP in all three samples were in the range of 78.2–100.2% as shown in Table 3, while matrix effect (n = 5) was observed between 92.20 and 101.35% in all three complex samples as given in Table 2.

**Table 3:** Contents and recovery studies of CAP in milk, human serum and urine samples at two fortification level 0.48 mg/kg and 5 mg/kg.

Sample	Analyte	Added (mg/kg)	Found (mg/kg)	Recovery (%)
Milk	CAP	0	0.00	0.00
		0.48	0.46	95 ± 14.23
		5	0.43	86.0 ± 14.23
Serum	CAP	00	0.00	0.00
		0.48	5.01	100.2 ± 11.28
		5	4.69	93.7 ± 9.23
Urine	CAP	00	0.00	0.00
		0.48	4.67	93.5 ± 14.96
		5	3.90	78.2 ± 13.69

(n = 5).

### The significance of the Method

The developed method is very attractive, cheap, green and facile as:

(a) It avoids the potential health risk imparted by pre/ post-column, electrochemical and other complex derivatization methods (avoids the need of such as, UV reactor, extra pump and reagents etc.); (b) green and cost effective as it minimized the use of reagents and chemicals; (c) utilizing the dual role of sorbent; (d) the method proved more selective and sensitive than previously developed method as shown in comparison Table 4; (e) involve µ-sample pretreatment; (f) inception of a new research area for determination of non-fluorescent compounds like CAP and *p*-nitophenol (*p*-NP). The fluorescent spectra of reduced non-fluorescent *p*-NP is given in Fig. 4(e).

**Table 4:** Comparison with reported methods in literature.

No.	Samples	Extraction methods	Analytical techniques	LOD	Ref.
1	Bulk drug and pharmaceutical dosage	SPE	HPLC-UV	1.4 mg/l	^59^
2	Plasma	SPE	UV spectrophotometry	0.5 mg/l	^60^
3	Milk	packed-nanofiber SPE	HPLC- UV	0.2 µg/l	^61^
4	Standard	SPE-MIP	FIA - fluorimetric detection	8000 µg/l	^29^
5	Pharmaceutical eye drops	SPE	SIA-HPLC with UV detection	500 µg/l	^6^
6	Milk, human serum and urine	µ-extraction method	IC-FLD	0.020 µg/kg	This method

FIA: flow injection analysis, SIA: sequential injection analysis, Photo-Diode Array (PDA) detector, SPE-MIP: solid-phase extraction with molecularly imprinted polymer.

## Conclusions

This work introduces a novel dual role of synthesized porous SnO_2_ nanoparticles in IC for the transformation of non-fluorescent antibiotic CAP and p-nitro phenol into their respective fluorescent products and as porous sorbent in the µ-QuEChERS based sample preparation method for riddance and degradation of interference and matrices in various complex samples (milk, human serum and urine samples).

### Instrumentation and Methods

#### Instrumentation

The IC-FLD separation and analysis were performed by using an Ion Chromatographic system (Thermo Fisher (ICS-1500 Waltham, MA, USA). An isocratic separation was conducted by using Ion Pac^®^ AS12A column (250 mm × 4 mm i.d; 13 mm particle size) protected by a guard column Ion Pac^®^ AG12A (50 mm × 4 mm i.d; 13 mm particle size) and sensitive detection was conducted by using a fluorescence detector (Ultimate 3000 RS, Dionex, Sunny vale, CA, USA). Furthermore, output signals were accumulated and subsequently processed with Chromeleons 7.2 software. The ultrasonic cleaner (SB-5200DT, Scientz Biotechnology Co. Ltd., Ningbo, China) was utilized for agitation, sonication and cleaning of apparatus and samples. The various characterization of nanoparticles were performed by using powder X-ray diffraction (XRD) method using Phillips X’Pert PRO diffractometer, scanning electron microscopy (SEM), transmission electron microscopy (TEM) images were obtained by using Hitachi SU-70 (Hitachi Japan), and H-7650 (Hitachi Japan), respectively. Fourier transform infrared (FTIR) spectroscopy was performed on SGE/Agilent 6890/Nicolet 5700 spectrometer (USA).

## Methods

Analytical standards of chloramphenicol (CAP), (≥98%) were purchased from Aladdin industrial Co. Ltd. (Shanghai, China), while HPLC-grade acetonitrile (ACN) and methanol were bought from Huipu Co. Hangzhou, China. Various NPs synthesis and mobile preparation reagents like sodium hydroxide, pure (50% in water), glycine (99.5%), magnesium sulfate (99.0%), tin (II) chloride dihydrate (≥99.0%) all were also purchased from Aladdin industrial Co. Ltd., Shanghai, China.

The selected real milk sample was purchased from the local market wall mart and stored in amber glass bottles. The real samples of human urine and serum were collected from the healthy volunteer from the local Zhejiang University medical hospital (yuquan branch). All the samples vials and bottles were wrapped in aluminum foil and kept at 4^o^C in the laboratory refrigerator. The informed consent of all volunteers being obtained for analysis of CAP in their samples. Both types of experiments (i) synthesis of SnO_2_ and (ii) analysis of CAP in biological samples were precisely performed in compliance of relevant local government and institution guidelines (Hangzhou Municipal Centre for Disease Control and Prevention Ethical Review Committee, China). Finally, all experimental protocols were approved by the analytical chemistry department of Zhejiang University.

### Synthesis of porous SnO_2_ NPs

The simple and completely green chemical precipitation method was used for the SnO_2_ NPs synthesis by following A. Bhattacharjee *et al*.^52^ scheme with slight modifications. Briefly, 0.02 M aqueous solution of SnCl_2_.2H_2_O and glycine were individually prepared in respective 100 ml flask and were subsequently mixed into a 400 or 500 ml clean beaker. The prepared 40 ml of 60 mmole SDS surfactant was added dropwise in the SnCl_2_.2H_2_O and glycine mixture with regular stirring at ambient temperature. Afterwards, the mixture was heated at 100 °C for 3–4 hours with continuous stirring until it turned yellow. Next day, the obtained precipitates were centrifuged, rinsed thrice with deionized water, dried at 60 °C and subsequently calcined at 600 °C for two hours. The obtained dry NPs powder was characterized and kept in a bottle for further use in the research work.

### µ-sample preparation method

**Figure 6:**
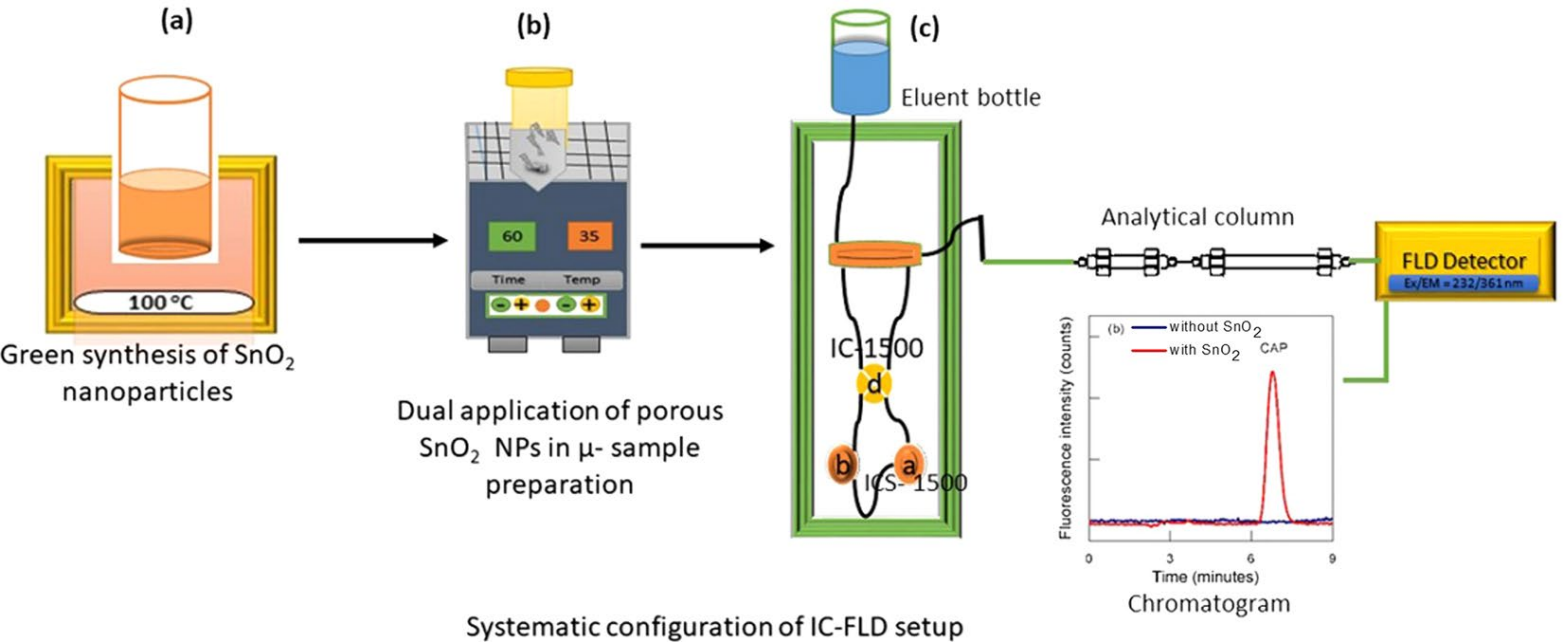
Systematic layout of dual application of porous SnO_2_ NPs in ion chromatography for determination of non-fluorescent CAP.

A novel µ- QuEChERS based sample preparation method was utilized for the transformation of the non-fluorescent antibiotic CAP into a fluorescent specie and its extraction from milk, human urine and serum samples. The optimized µ-sample preparation extraction method involved conditioning of porous 60 µg SnO_2_ sorbent in 4.0 ml centrifuge tube; meanwhile, 300 µl of each NaBH_4_ and sample (milk, human urine and serum) were mixed and fortified with 0.48 mg/kg CAP in another similar 4.0 ml centrifuge vial. The resulting mixture was vigorously shaken for a moment before it was poured into sorbent tube. The mixture was sonicated for about 60 minutes at a temperature in the range of 55–60 °C to convert non-fluorescent CAP into its fluorescent form and for maximum decomposition and adsorption of matrices/ interferences (organic, inorganic and polar matrixes) on the porous sorbent. Afterward, the sonicated mixture was precisely centrifuged at speed of 9500 RPM for 5 minutes to halt the reaction. Then, a mixture of 140 µg MgSO_4_, 70 µg NaCl and later 900 µl HPLC grade ACN was added and put on sonication bath for another 10 minutes in order to obtain maximum reduced CAP back-extraction in the acetonitrile solvent and to disperse polar and inorganic impurities/matrices in the aqueous layer. Finally, it was again centrifuged at the same speed for 12 minutes to settle down the used nanoparticles as a sorbent. In between two layers appeared, the upper organic layer containing CAP was precisely poured into another clean vial and it was dried with the help of gentle inert N_2_ gas stream. Finally, dried residue was reconstituted to 900 µl with mobile phase and injected into the IC-FLD system with 1 mL syringe having 0.22 µm membrane filter for CAP sensitive fluorescence analysis. The same procedure was adopted for all selected samples, except real human serum which was first diluted 10 times with acetonitrile in order to homogenize the human serum and to precipitate the protein. Systematic SnO_2_ nanoparticles synthesis and its dual role in the µ-sample preparation and IC-FLD analysis is illustrated in Fig. 6.

### Method validation

To investigate the liability of the proposed novel method, it was completely validated in term of selectivity, linearity, detection limit (LOD), quantification limit (LOQ), matrix effect, accuracy and intra- and inter-day precision.

Selectivity of the method was investigated by observing any interfering peaks at the retention time of CAP. Five points calibration curve (0.48, 1.5, 3 and 5 mg/kg) was plotted for linearity and estimation of matrix effect. The calibration curve in the pure solvent was prepared by spiking the CAP respective concentration in 50% ACN: H_2_O, and then went through µ-sample treatment, while matrix-matched calibration curve was prepared by spiking CAP into 300 µl milk, human serum and urine sample and followed the same step as pure solvent spiked calibration. The matrix effect was evaluated by comparing CAP fluoresce response obtained in ACN solvent and respective matrices. ME was calculated by applying the following formula:

$${\rm{ME}}( \% )={\rm{B}}/{\rm{A}}\times 100,$$

where each A and B are the peak areas of CAP in matrix extract and pure solvent, respectively. Both LOD and LOQ were estimated based on the injected concentration at which signal to noise ratio (S/N) equal to 3 and 10-fold, respectively. Furthermore, intra- and inter-day precision were calculated within one and over three consecutive days and every injection was repeated in the quintet.

## Electronic supplementary material

Supplementary Information
