# Supplementary material for: Porous SnO2 nanoparticles based ion chromatographic determination of non-fluorescent antibiotic (chloramphenicol) in complex samples
Source: Sci Rep. 2018 Aug 17;8:12327. doi: 10.1038/s41598-018-29922-5 (PMC6098012; doi:10.1038/s41598-018-29922-5)
Supplement: Supplementary file 1 — Supplementary Information [file 41598_2018_29922_MOESM1_ESM.docx]

**Porous Sno_2_ nanoparticles based ion chromatographic determination of non-fluorescent antibiotic (chloramphenicol) in complex samples**

Nadeem Muhammad^1, 2^, Abdul Rahman^2^, Muhammad Adnan Younis^2^, Qamar Subhani^2^, Khurram Shehzad^3*^, [Hairong Cui](http://pubs.rsc.org/en/results?searchtext=Author%3AHairong%20Cui)^1^, Yan Zhu^2*^

^1^Department of Environmental Engineering, Wuchang University of Technology, Wuhan, China.

^2^ Department of Chemistry, Zhejiang University, Hangzhou 310028, China.

^3*^Department of IT and Electronics, 310027, Hangzhou, China.

(*) Corresponding author:

Tel.: +86 571 88273637 Fax: +86 571 88823446.

*E-mail address*: [khurrams@zju.edu.cn](mailto:khurrams@zju.edu.cn) (K. Shehzad); zhuyan@zju.edu.cn (Y. Zhu)

**Supplementary Information**

**Contents**

Figure S-1– Average particles size of 50 random SnO2 NPs with RSD bar………………………………...............................1

Figure S-2– Fluorescence emission and excitation spectra of reduced CAP ……………………………….............................. 1 Figure S-3 (a) – Comparison of effect of different temperature on fluorescence intensity of CAP.................................... 2

Figure S-3 (b) – Comparison of effect of different extracting solvents on fluorescence intensity of CAP…………………………….. 2

**Fig. S-1** Average particles size of 50 random SnO2 NPs with RSD bar.

**Fig. S-2** (a) The fluorescence excitation and emission spectra of sorbent treated CAP in basic media.

**Fig. S3 (a)** Comparison of effect of different temperature on fluorescence intensity of CAP and (**b)** Comparison of effect of different extracting solvents on fluorescence intensity of CAP.
